# Impact of the endoscopic surgical skill qualification system on conversion to laparotomy after low anterior resection for rectal cancer in Japan (a secondary analysis of the EnSSURE study)

**DOI:** 10.1007/s00464-024-10740-y

**Published:** 2024-03-08

**Authors:** Koki Goto, Jun Watanabe, Toshiya Nagasaki, Mamoru Uemura, Heita Ozawa, Yohei Kurose, Tomonori Akagi, Nobuki Ichikawa, Hiroaki Iijima, Masafumi Inomata, Akinobu Taketomi, Takeshi Naitoh, EnSSURE study group collaboratives in Japan Society of Laparoscopic Colorectal Surgery, Akinobu Furutani, Akiyoshi Kanazawa, Akiyoshi Noda, Atsushi Ishibe, Chikayoshi Tani, Daisuke Yamamoto, Fumihiko Fujita, Fuminori Teraishi, Fumio Ishida, Fumitaka Asahara, Hideaki Karasawa, Hideki Osawa, Hiroaki Nagano, Hiroaki Takeshita, Hirofumi Ota, Hirokazu Suwa, Hiroki Ochiai, Hiroomi Ogawa, Hiroshi Saeki, Hirotoshi Hasegawa, Hiroyuki Bando, Hisanaga Horie, Hisashi Nagahara, Kaori Hayashibara, Kay Uehara, Kazuhiro Takehara, Ken Kojo, Ken Okamoto, Kenichiro Saito, Koji Ikeda, Koji Munakata, Koki Otsuka, Koya Hida, Kunihiko Nagakari, Manabu Shimomura, Manabu Shiozawa, Manabu Takata, Manabu Yamamoto, Masaaki Ito, Masakatsu Numata, Masahiko Watanabe, Masashi Miguchi, Mayumi Ozawa, Mitsuhisa Takatsuki, Naoya Aisu, Naruhiko Sawada, Nobuaki Suzuki, Ryo Ikeshima, Ryo Inada, Ryuichi Oshima, Satoshi Maruyama, Shigehiro Kojima, Shigeki Yamaguchi, Shigenori Homma, Shiki Fujino, Shinichiro Mori, Shinobu Ohnuma, Sho Takeda, Shota Aoyama, Shuji Saito, Shunpei Mukai, Shusaku Takahashi, Takahiro Sasaki, Takahiro Yamanashi, Takeru Matsuda, Takuya Miura, Tatsunari Fukuoka, Tatsunori Ono, Tatsuya Kinjo, Tatsuya Shonaka, Teni Godai, Tohru Funakoshi, Tomohiro Adachi, Tomohiro Yamaguchi, Tomohisa Furuhata, Toshimoto Kimura, Toshisada Aiba, Toshiyoshi Fujiwara, Tsukasa Shimamura, Tsunekazu Mizushima, Yasuhito Iseki, Yasuo Sumi, Yasushi Rino, Yasuyuki Kamada, Yoshiaki Kita, Yoshihiro Kakeji, Yoshihiro Takashima, Yoshihito Ide, Yoshiharu Sakai, Yoshinori Munemoto, Yoshito Akagi, Yoshiyuki Ishii, Yuji Inoue, Yuki Kiyozumi, Yukihito Kokuba, Yukitoshi Todate, Yusuke Suwa, Yusuke Sakimura, Yusuke Shimodaira

**Affiliations:** 1 Department of Surgery, Gastroenterological Center, Yokohama City University Medical Center, Yokohama, Japan; 2 Department of Gastroenterological Surgery, Cancer Institute Hospital of the Japanese Foundation for Cancer Research, Tokyo, Japan; 3 Department of Gastroenterological Surgery, Graduate School of Medicine, Osaka University, Suita, Japan; 4 Department of Colorectal Surgery, Tochigi Cancer Center, Utsunomiya, Japan; 5 Department of Surgery, Fukuyama City Hospital, Fukuyama, Japan; 6 Department of Gastroenterological and Pediatric Surgery, Oita University, Oita, Japan; 7 Department of Gastroenterological Surgery I, Graduate School of Medicine, Hokkaido University, Sapporo, Japan; 8 Department of Lower Gastrointestinal Surgery, Kitasato University School of Medicine, Sagamihara, Japan

**Keywords:** Rectal cancer, Laparoscopic surgery, Rectal resection, Conversion, Japan, Endoscopic surgical skill qualification system

## Abstract

**Background and aims:**

Conversion to laparotomy is among the serious intraoperative complications and carries an increased risk of postoperative complications. In this cohort study, we investigated whether or not the Endoscopic Surgical Skill Qualification System (ESSQS) affects the conversion rate among patients undergoing laparoscopic surgery for rectal cancer.

**Methods:**

We performed a retrospective secondary analysis of data collected from patients undergoing laparoscopic surgery for cStage II and III rectal cancer from 2014 to 2016 across 56 institutions affiliated with the Japan Society of Laparoscopic Colorectal Surgery. Data from the original EnSSURE study were analyzed to investigate risk factors for conversion to laparotomy by performing univariate and multivariate analyses based on the reason for conversion.

**Results:**

Data were collected for 3,168 cases, including 65 (2.1%) involving conversion to laparotomy. Indicated conversion accounted for 27 cases (0.9%), while technical conversion accounted for 35 cases (1.1%). The multivariate analysis identified the following independent risk factors for indicated conversion to laparotomy: tumor diameter [mm] (odds ratio [OR] 1.01, 95% confidence interval [CI] 1.01–1.05, *p *= 0.0002), combined resection of adjacent organs [+/−] (OR 7.92, 95% CI 3.14–19.97, *p *< 0.0001), and surgical participation of an ESSQS-certified physician [−/+] (OR 4.46, 95% CI 2.01–9.90, *p *= 0.0002). The multivariate analysis identified the following risk factors for technical conversion to laparotomy: registered case number of institution (OR 0.99, 95% CI 0.99–1.00, *p *= 0.0029), institution type [non-university/university hospital] (OR 3.52, 95% CI 1.54–8.04, *p *= 0.0028), combined resection of adjacent organs [+/−] (OR 5.96, 95% CI 2.15–16.53, *p *= 0.0006), and surgical participation of an ESSQS-certified physician [−/+] (OR 6.26, 95% CI 3.01–13.05, *p *< 0.0001).

**Conclusions:**

Participation of ESSQS-certified physicians may reduce the risk of both indicated and technical conversion. Referral to specialized institutions, such as high-volume centers and university hospitals, especially for patients exhibiting relevant background risk factors, may reduce the risk of conversion to laparotomy and lead to better outcomes for patients.

**Trial Registration:**

This study was registered with the Japanese Clinical Trials Registry as UMIN000040645.

Several large-scale randomized controlled trials (RCTs) conducted in the 2010s demonstrated that laparoscopic surgery yielded better short-term outcomes for rectal cancer than laparotomy, without increasing rates of postoperative or surgical complications [1–4]. Recent studies have also reported a similar long-term prognosis between laparoscopic surgery and laparotomy in terms of the local recurrence-free survival and recurrence-free survival, and the oncological safety of both techniques has been demonstrated [5–8].

The first RCT to compare laparoscopic surgery and laparotomy for colorectal cancer reported that the rate of conversion to laparotomy in the laparoscopic surgery group was high, at 34% [9]. Despite subsequent decreases in the rate of conversion to laparotomy the recent COLOR II trial, which compared laparoscopic surgery and laparotomy for rectal cancer, reported a conversion rate of 16% [2], and some studies continue to report relatively high values.

Conversion to laparotomy is an extremely serious intraoperative complication that can occur in patients undergoing laparoscopic surgery, resulting in prolonged operation times, increased blood loss, prolonged postoperative hospitalization, and an increased risk of postoperative complications [10–12]. Additional studies have reported a poor overall survival in patients undergoing laparoscopic surgery for colon cancer [13], as well as a poor recurrence-free survival in patients undergoing laparoscopic surgery for rectal cancer [14]. Thus, decreasing the conversion to laparotomy rate may help improve both short- and long-term outcomes following laparoscopic surgery for rectal cancer.

Previous studies have identified the following as risk factors for conversion to laparotomy: age, sex, obesity, presence or absence of diverticular disease, a history of abdominal surgery, depth of tumor wall invasion, institution specialty and size, and the age/proficiency of surgeons [13, 15–23]. Given this finding concerning the age/proficiency of surgeons, the Japan Society for Endoscopic Surgery (JSES) introduced the Endoscopic Surgical Skill Qualification System (ESSQS) in 2004 to maintain the safety and quality of laparoscopic surgery and educate trainers in Japan, and they have provided technical accreditation for laparoscopic surgery since the inception of the program. ESSQS-certified surgeons are certified as not only skilled operators but also instructors based on the screening of surgical technique videos, and they have contributed substantially to the passing on and quality assurance of laparoscopic surgical techniques for the colon [24–26]. Several small-scale studies have demonstrated better clinical outcomes for surgeries supervised by an ESSQS-certified physician than those without such supervision [27–29]; however, hardly any studies have used large-scale data to evaluate the efficacy and usefulness of ESSQS certification.

Therefore, in the present study, we accumulated data from 56 institutions belonging to the Japan Society of Laparoscopic Colorectal Surgery (JSLCS) to examine the impact of the surgical participation of an ESSQS-certified physician on the conversion to laparotomy rate. We also examined other risk factors influencing conversion to laparotomy in patients undergoing laparoscopic rectal resection.

## Materials and methods

We targeted patients who underwent laparoscopic surgery for rectal cancer from January 2014 to December 2016 at 56 institutions affiliated with JSLCS. For the current study, we performed a secondary analysis of data collected for the EnSSURE study (The Study investigating the Impact of Endoscopic Surgical Skill Qualification in Laparoscopic Resection for Rectal Cancer in Japan) [30]. The study protocol was approved by the Institutional Review Board of Hokkaido University Hospital and each participating hospital prior to initiation of the study, and it was registered in the Japanese Clinical Trials Registry on June 3, 2020 (UMIN000040645; http://www.umin.ac.jp/ctr/index.htm). Due to the retrospective nature of the study, written informed consent was not obtained. An opt-out method was used to disclose information about the study.

Patient data were collected from clinical reports. The eligibility criteria were as follows: (1) rectal and rectosigmoid tumor, (2) a histological diagnosis of rectal cancer, (3) clinical stage II and III, and (4) elective surgery. The exclusion criteria were as follows: (1) synchronous or metachronous multicentric cancers or multiple cancers within five years, (2) other surgeries performed at the same time, (3) robot-assisted surgery, (4) ulcerative colitis, (5) cases of total colectomy and total pelvic exenteration, and (6) cases judged as inappropriate by the investigator. Demographic and clinicopathological data, including the presence or absence of conversion to laparotomy, were collected and analyzed in a retrospective manner.

The impact of the ESSQS on conversion to laparotomy after low anterior resection for rectal cancer were considered the primary endpoint. Conversion to laparotomy was defined as a case requiring a skin incision of ≥8 cm, and these cases were divided into 2 types based on the reason for conversion. “Indicated conversion” was defined as cases in which severe infiltration into other organs, distant metastasis, or multiple cancers was found during surgery, and in which laparoscopic surgery was judged to be inappropriate, leading to laparotomy. “Technical conversion” was defined as laparotomy due to technical factors such as operator concerns and control of intraoperative complications (intra-abdominal bleeding, organ damage, etc.) [31]. The concept is a very important concept that has been previously reported, and all of this information was judged from the description in the surgical records and classified strictly.

We analyzed the risk factors for these two types of conversion in the present study.

### The ESSQS by the JSES

Qualifications for taking the ESSQS-certified physician [24–26] test stipulated by the JSES include experience in laparoscopic surgery, participation in official JSES training seminars, and at least two years of experience as a general surgeon after becoming a Japan Surgical Society-certified physician. The test involves a random video review of anonymous and unedited videos, with skill judgments made by two or more JSES-stipulated laparoscopists. To become an ESSQS-certified physician in the colon department, surgeons must submit videos depicting sigmoid resection for sigmoid colon cancer or high anterior resection for rectosigmoid cancer. Acquisition of ESSQS certification is considered essential for the safe implementation of laparoscopic surgery in Japan, and the pass rate of examinees is about 20%–30% every year, with less than 10% of general surgeons in Japan currently falling under this category [30]. Since certification implies one’s skill as both an operator and instructor [27, 28], cases in which the physician participated in the surgery as an operator, assistant, or instructor were all classified as “surgical participation by an ESSQS-certified physician” in this study.

### Statistical analyses

Data were presented as means and standard deviations or as numbers and percentages, as appropriate. The risk factors for indicated conversion and technical conversion were analyzed separately. We first performed univariate regression analysis to select variables, which showed the p-value less than 0.05, and then the multiple logistic regression was performed using those selected variables to obtain the adjusted Odds ratios (OR). OR and their 95% confidence intervals (CIs) were calculated using a multivariate logistic regression analysis.

All statistical analyses were conducted using the JMP® Pro software program, ver. 16.1 (JMP Statistical Discovery LLC, Cary, North Carolina, USA). P values were considered statistically significant at *p*<0.05 (2-sided).

## Results

**Fig. 1:**
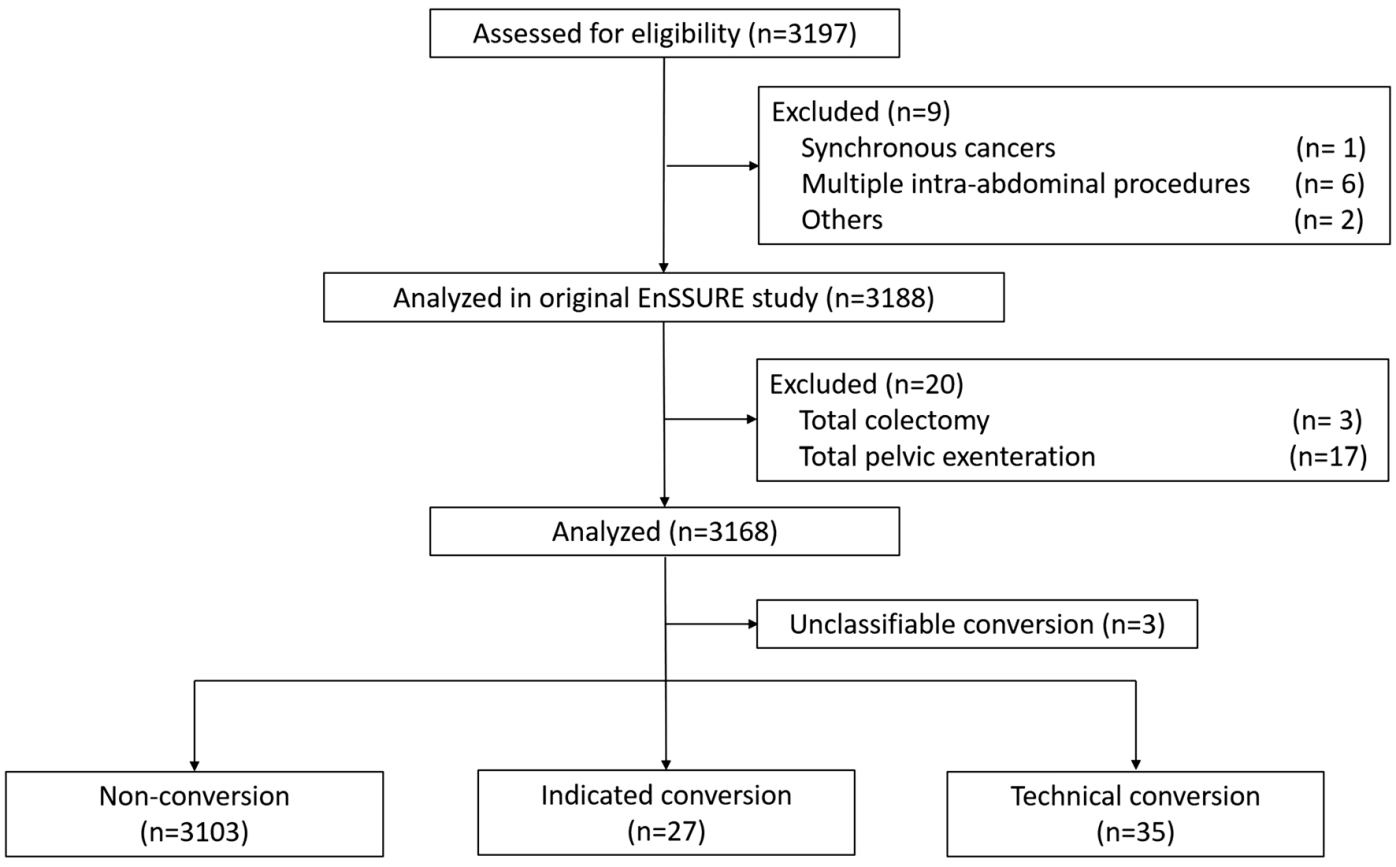
Flow diagram of patient disposition

Figure 1 shows the flow diagram for patient selection. Data were collected for 3168 patients who underwent laparoscopic surgery for rectal cancer between 2014 and 2016 across 56 institutions participating in the JSLCS. Laparoscopic conversions accounted for 65 cases (2.1%), including indicated conversions in 27 cases (0.9%), technical conversions in 35 cases (1.1%), and unclassifiable conversions in 3 cases (0.1%).

Table 1 shows the background characteristics of the non-conversion, indicated conversion, and technical conversion groups. Factors evaluated included the age, sex, body mass index (BMI), American Society of Anesthesiologists physical status classification (ASA-PS), preoperative treatment, details of preoperative treatment, preoperative obstruction, tumor diameter (mm), tumor location (RS/Ra/Rb), cStage (II/III), cT (1/2/3/4a/4b), cN (N0/N1/N2/N3), surgical procedure (HAR/LAR/ISR/Hartmann/APR), diverting stoma, combined resection of adjacent organs, degree of lymph node dissection (D0/D1/D2/D3), inferior mesenteric artery high ligation, lateral lymph node dissection, mobilization of splenic flexion, registered case number of institution, institution type (non-university hospital/university hospital), and surgical participation of an ESSQS-certified physician.

**Table 1:** Patient’s background characteristics

	Non-conversion (*n *= 3103)	Indicated conversion (*n* = 27)	Technical conversion (*n* = 35)
Age (years)	64.5 (12.0)	60.9 (13.8)	65.4 (12.0)
Sex (*n*, %)			
Male	1960 (98.0)	21 (1.1)	18 (0.9)
Female	1143 (98.0)	6 (0.5)	17 (1.5)
BMI (kg/m^2^)	22.6 (3.5)	22.6 (3.9)	23.5 (3.8)
ASA (*n*, %)			
1	937 (97.5)	9 (0.9)	15 (1.6)
2	1870 (98.3)	15 (0.8)	17 (0.9)
3	227 (98.2)	2 (0.9)	2 (0.9)
4	3 (100)	0	0
Unknown	66 (97.1)	1(1.5)	1(1.5)
Preoperative treatment (*n*, %)			
+	722 (98.4)	7 (0.9)	5 (0.7)
−	2381 (98.0)	20 (0.8)	30 (1.2)
Detail of preoperative treatment (*n*, %)			
CRT	323 (98.8)	0	4 (1.2)
TNT	121 (99.2)	1 (0.8)	0
NAC	276 (97.5)	6 (2.1)	1 (0.4)
Others	2 (100)	0	0
None	2381 (98.0)	20 (0.8)	30 (1.2)
Preoperative obstruction (*n*, %)			
+	148 (95.5)	4 (2.6)	3 (1.9)
-	2955 (98.2)	23 (0.8)	32 (1.1)
Diameter of tumor (mm)	44.0 (18.5)	62.1 (19.4)	49.8 (15.7)
Tumor location (*n*, %)			
RS	972 (97.6)	11 (1.1)	13 (1.3)
Ra	1004 (97.6)	11 (1.1)	14 (1.3)
Rb	1122 (98.9)	5 (0.4)	8 (0.7)
Unknown	5(100)	0	0
cStage (*n*, %)			
II	1407 (98.7)	7 (0.5)	11 (0.8)
III	1696 (97.5)	20 (1.1)	24 (1.4)
cT (*n*, %)			
1	36 (100)	0	0
2	161 (100)	0	0
3	2313 (98.8)	9 (0.4)	19 (0.8)
4a	438 (94.6)	11 (2.4)	14 (3.0)
4b	153 (94.5)	7 (4.3)	2 (1.2)
Unknown	2 (100)	0	0
cN (*n*, %)			
N0	1407 (98.7)	7 (0.5)	11 (0.8)
N1	1174 (98.0)	8 (0.7)	16 (1.3)
N2	347 (96.1)	8 (2.2)	6 (1.7)
N3	175 (96.7)	4 (2.2)	2 (1.1)
Surgical procedure (*n*, %)			
HAR	610 (97.9)	6 (1.0)	7 (1.1)
LAR	1768 (97.9)	17 (0.9)	21 (1.2)
ISR	197 (98.0)	2 (1.0)	2 (1.0)
Hartmann	99 (96.1)	2 (1.9)	2 (1.9)
APR	429 (99.3)	0	3 (0.7)
Diverting Stoma (*n*, %)			
+	1013 (98.0)	13 (1.2)	8 (0.8)
-	2090 (98.0)	14 (0.7)	27 (1.3)
Combined resection of adjacent organs (*n*, %)			
+	121 (89.6)	9 (6.7)	5 (3.7)
-	2982 (98.4)	18 (0.6)	30 (1.0)
LN dissection (*n*, %)			
D0	5 (100)	0	0
D1	7(100)	0	0
D2	265 (99.2)	1 (0.4)	1 (0.4)
D3	2826 (97.9)	26 (0.9)	34 (1.2)
IMA high ligation (*n*, %)			
+	2296 (98.2)	22 (0.9)	22 (0.9)
-	803 (97.8)	5 (0.6)	13 (1.6)
Unknown	4 (100)	0	0
Lateral LN dissection (*n*, %)			
+	554 (97.7)	6 (1.1)	7 (1.2)
−	2549 (98.1)	21 (0.8)	28 (1.1)
Mobilization of SF (*n*, %)			
+	277 (97.5)	4 (1.4)	3 (1.1)
-	2763 (98.7)	19 (0.7)	18 (0.6)
Unknown	63 (77.8)	4 (4.9)	14 (17.3)
Registered case number of the institution			
	127 (131)	99 (86)	67 (50)
Institution type (*n*, %)			
Not University hospital	1612 (97.2)	19 (1.2)	27 (1.6)
University hospital	1491 (99.0)	8 (0.5)	8 (0.5)
ESSQS (*n*, %)			
+	2596 (98.9)	15 (0.6)	13 (0.5)
−	507 (93.7)	12 (2.2)	22 (4.1)

*BMI* body mass index, *ASA* American Society of Anesthesiologists, *CRT* chemoradiation therapy, *TNT* total neoadjuvant therapy, *NAC* neoadjuvant therapy, *HAR* high anterior resection, *LAR* low anterior resection, *ISR* intersphincteric resection, *APR* abdominoperineal resection, *TC* total colectomy, *IMA* inferior mesenteric artery, *SF* splenic flexure, *ESSQS* the endoscopic surgical skill qualification system

Table 2 shows the details of the reason for conversion to laparotomy for indicated conversion and technical conversion. The majority of indicated conversion were associated with tumor invasion of other organs or extended resection in 14 cases (51.9%), and giant tumor in 11 cases (40.7%). In one case of indicated conversion, the reason for conversion to laparotomy was due to tumor progression, but the details were unclear. Technical conversion was associated with failure to expand the field of vision in 14 cases (40.0%), injury to other organs in 6 cases (17.1%), adhesions in 5 cases (14.3%), bleeding in 5 cases (14.3%), and anastomotic problems in 5 cases (14.3%).

**Table 2:** Details of reasons for conversion to laparotomy

Indicated conversion (*n *= 27)	
Invasion of other organs/extended resection	14 (51.9)
Giant tumor	11 (40.7)
Lateral LN dissection	1 (3.7)
Others	1 (3.7)
Technical conversion (*n *= 35)	
Failure to expand the field of vision	14 (40.0)
Injury to other organs	6 (17.1)
Adhesions	5 (14.3)
Bleeding	5 (14.3)
Anastomotic problems	5 (14.3)

Table 3 shows the results of the univariate and multivariate analysis of risk factors for indicated conversion. The univariate analysis extracted the following five factors as risk factors for indicated conversion: preoperative obstruction [+/−] (OR 3.47, 95% CI 1.19–10.17, *p *= 0.0232), tumor diameter (mm) (OR 1.04, 95% CI 1.02–1.06, *p *< 0.0001), cT [cT3/shallower than T3] (OR 8.49, 95% CI 3.80–19.00, *p *< 0.0001), combined resection of adjacent organs [+/−] (OR 11.72, 95% CI 5.16–26.59, *p *< 0.0001), and surgical participation of an ESSQS-certified physician [+/−] (OR 4.10, 95% CI 1.91–8.80, *p *= 0.0003). Of the five factors that were risk factors in the multivariate analysis, the T factor and combined resection of adjacent organs overlapped. Therefore, the multivariate analysis was conducted with four factors, omitting the T factor. The following 3 factors were extracted as independent risk factors for indicated conversion: tumor diameter (mm) (OR 1.01, 95% CI 1.01–1.05, *p *= 0.0002), combined resection of adjacent organs [+/−] (OR 7.92, 95% CI 3.14–19.97, *p *< 0.0001), and surgical participation of an ESSQS-certified surgeon [+/−] (OR 4.46, 95% CI 2.01–9.90, *p *= 0.0002).

**Table 3:** Risk factors for indicated conversion

	Univariate analysis			Multivariate analysis		
	OR	*p* value	95%CI	OR	*p* value	95%CI
Age (years)	0.98	0.1227	0.95–1.01			
Sex [male/female]	2.04	0.1245	0.82–5.07			
BMI (kg/m^2^)	1.00	0.9410	0.90–1.12			
ASA [class 2/class 1]	0.84	0.6705	0.36–1.92			
ASA [class 3/class 1]	0.92	0.9124	0.20–4.27			
Preoperative treatment [+/−]	1.15	0.7445	0.49–2.74			
Preoperative obstruction [+/−]	3.47	0.0232	1.19–10.17	1.47	0.5358	0.44–4.95
Diameter of tumor (mm)	1.04	<0.0001	1.02–1.06	1.01	0.0002	1.01–1.05
Tumor location [Ra/RS]	0.97	0.9398	0.42–2.24			
Tumor location [Rb/RS]	0.39	0.0850	0.14–1.14			
cT [cT4/shallower than T3]	8.49	<0.0001	3.80–19.00			
cN [N+/N−]	2.37	0.0502	1.00–5.62			
Surgical procedure [with anastomosis/without anastomosis]	2.56	0.2012	0.61–10.85			
Combined resection of adjacent organs [+/−]	11.72	<0.0001	5.16–26.59	7.92	<0.0001	3.14–19.97
LN Dissection [D3/less than D2]	2.55	0.3595	0.34–18.85			
IMA high ligation [+/−]	1.54	0.3878	0.58–4.07			
Mobilization of SF [+/−]	2.10	0.1800	0.71–6.21			
Lateral LN dissection [+/−]	1.31	0.5566	0.53–3.27			
Registered case number of the institution	0.99	0.2268	0.99–1.00			
Institution type [not University hospital/University hospital]	2.20	0.0628	0.96–5.03			
ESSQS [−/+]	4.10	0.0003	1.91–8.80	4.46	0.0002	2.01–9.90

*ASA* American Society of Anesthesiologists, *IMA* inferior mesenteric artery, *SF* splenic flexure, *ESSQS* the endoscopic surgical skill qualification system, *OR* odds ratio, *CI* confidence interval

Table 4 shows the results of the univariate and multivariate analysis of risk factors for technical conversion. The univariate analysis extracted the following five factors as risk factors for technical conversion: cT [cT4/shallower than T3] (OR 3.58, 95% CI 1.83–7.00, *p *= 0.0002), combined resection of adjacent organs [+/−] (OR 3.90, 95% CI 1.49–10.23, *p *= 0.0056), registered case number of institution (OR 0.99, 95% CI 0.99–1.00, *p *= 0.0010), institution type [non-university/university hospital) (OR 3.12, 95% CI 1.41–6.89, *p *= 0.0049), and surgical participation of an ESSQS-certified surgeon [−/+] (OR 8.73, 95% CI 4.34–17.31, *p *< 0.0001). Of the five factors that were risk factors in the multivariate analysis, the T factor and the combined resection of adjacent organs overlapped, so the multivariate analysis was conducted with four factors, omitting the T factor. The following 4 factors were extracted as independent risk factors for technical conversion: combined resection of adjacent organs [+/−] (OR 5.96, 95% CI 2.15–16.53, *p *= 0.0006), registered case number of institution (OR 0.99, 95% CI 0.99–1.00, *p *= 0.0029), institution type [non-university/university hospital] (OR 3.52, 95% CI 1.54–8.04, *p *= 0.0028), and surgical participation of ESSQS-certified physician [−/+] (OR 6.26, 95% CI 3.01–13.05, *p *< 0.0001).

**Table 4:** Risk factors for technical conversion

	Univariate analysis			Multivariate analysis		
	OR	*p* value	95%CI	OR	*p* value	95%CI
Age (years)	1.01	0.6511	0.99–1.04			
Sex [male/female]	0.62	0.1565	0.32–1.20			
BMI (kg/m^2^)	1.07	0.1495	0.98–1.17			
ASA [class 2/class 1]	0.57	0.1125	0.28–1.14			
ASA [class 3/class 1]	0.55	0.4298	0.12–2.42			
Preoperative treatment [+/−]	0.55	0.2171	0.21–1.42			
Preoperative obstruction [+/−]	1.87	0.3038	0.57–6.18			
Diameter of tumor (mm)	1.02	0.0742	0.98–1.03			
Tumor location [Ra/RS]	1.04	0.9143	0.49–2.23			
Tumor location [Rb/RS]	0.53	0.1636	0.22–1.29			
cT [cT4/shallower than T3]	3.58	0.0002	1.83–7.00			
cN [N+/N−]	1.81	0.1049	0.88–3.71			
Surgical procedure [with anastomosis/without anastomosis]	1.23	0.6694	0.48–3.19			
Combined resection of adjacent organs [+/−]	3.90	0.0056	1.49–10.23	5.96	0.0006	2.15–16.53
LN dissection [D3/less than D2]	3.33	0.2363	0.45–24.43			
IMA high ligation [+/−]	0.59	0.1352	0.30–1.18			
Mobilization of SF [+/−]	1.66	0.4174	0.49–5.68			
Lateral LN dissection [+/−]	1.15	0.7419	0.50–2.65			
Registered case number of the institution	0.99	0.0010	0.99–1.00	0.99	0.0029	0.99–1.00
Institution type [not University hospital/University hospital]	3.12	0.0049	1.41–6.89	3.52	0.0028	1.54–8.04
ESSQS [−/+]	8.73	<0.0001	4.34–17.31	6.26	<0.0001	3.01–13.05

*ASA* American Society of Anesthesiologists, *IMA* inferior mesenteric artery, *SF* splenic flexure, *ESSQS* the endoscopic surgical skill qualification system, *OR* odds ratio, *CI* confidence interval

## Discussion

Initial RCTs reported relatively high rates of conversion to laparotomy among patients undergoing laparoscopic surgery for colon cancer, with values of 21% in the COST study [32], 29% in the CLASSIC study [9], and 19% in the COLOR study [33]. However, reported conversion rates have decreased over time [13], with the recent JCOG0404 study from Japan reporting a rate of 5.5% [31]. Although surgery for rectal cancer is more difficult than for colon cancer, the conversion to laparotomy rates for rectal procedures in recent large-scale RCTs were 16% in the COLOR II study [2], 11% in the ACOSOG Z6051 study [3], 9% in the ALaCaRT study [4], and 1.7% in the COREAN study [1]. The conversion to laparotomy rate in the current study was 2.0%, which is relatively low when compared with the rates reported in these previous RCTs. We assessed the risk factors for conversion to laparotomy based on the reason for conversion and notably demonstrated that the participation of an ESSQS-certified surgeon in the surgical team reduced the risks for both indicated and technical conversions after laparoscopic surgery for rectal cancer. The present result indicated that the participation of an ESSQS-certified surgeon in the surgical team helped avoid conversion by overcoming both oncological and technical difficulties during laparoscopic surgery for rectal cancer.

In a recent review comparing long-term oncological outcomes between a laparoscopic surgery completion group and a conversion to laparotomy group in cases of laparoscopic surgery for colorectal cancer, three studies reported a significant difference in the overall survival (OS) [34–36], and five studies reported a significant difference in the disease-free survival (DFS) [34, 36–39], with all reports demonstrating a poor prognosis in the conversion to laparotomy group. However, several studies have indicated that the BMI, tumor diameter, and tumor stage are greater in the conversion to laparotomy group than the laparoscopic surgery completion group [10, 11, 34–37, 40, 41]. As these factors may all exert a negative effect on oncological outcomes and the survival, conversion to laparotomy itself may not be a predictor of a poor prognosis.

However, Furnée et al. reported that conversion to laparotomy was a significant predictor of the DFS, independent of other factors that were included in the multivariate analysis [14], suggesting a direct impact of conversion to laparotomy on the DFS. Given its high degree of surgical invasiveness, conversion to laparotomy induces an inflammatory reaction and decreased antitumor immunity, which results in an increased risk of distant metastasis; this is in turn thought to exert a negative influence on oncological outcomes [42]. Therefore, avoiding conversion to laparotomy may help improve the long-term prognosis among patients undergoing laparoscopic surgery for rectal cancer.

Avoiding conversion to laparotomy requires an understanding of relevant risk factors and the implementation of appropriate countermeasures. Various background characteristics have been identified as risk factors for conversion to laparotomy in previous studies, including age, sex, obesity, and diverticular disease, as well as a history of abdominal surgery and adhesion [13, 15–18]. Similarly, oncological background characteristics, including the tumor diameter, tumor wall invasion depth T4, and tumor stage, have been cited as risk factors for conversion [15, 36]. In the present study, oncological background characteristics were a risk factor for indicated conversion, as were surgical factors, such as the tumor diameter and combined resection of adjacent organs. Combined resection was also identified as a risk factor for technical conversion. Necessary countermeasures for these risk factors include an accurate preoperative diagnosis and sufficient examination of indications as to whether or not laparoscopic surgery can be completed at individual institutions and by specific surgical members. Studies have also reported that the long-term prognosis is worse for cases in which the decision to convert to laparotomy is made during laparoscopic surgery than when laparotomy is originally planned [9]. Depending on the case, selecting laparotomy from the start may reduce the likelihood of conversion to laparotomy, which may help improve both the short-term and long-term prognosis.

In the present study, the registered case number at each institution and whether or not it was a university hospital were extracted as risk factors for technical conversion. The registered case number represents the number of laparoscopic surgeries for rectal cancer performed during the same period, meaning that there were many technical conversions at small institutions despite a small number of surgeries. Conversely, there were few technical conversions at university hospitals, where colorectal surgery is expected to be conducted by proficient operators. Previous research has indicated that specialization in colorectal surgery, an individual’s or institution’s expertise in colorectal surgery, and the scale of the institution are risk factors relevant to conversion to laparotomy [23]. The result of this study, similar to previous reports, showed that high-quality surgeries were performed in the highly specialized institution in colorectal surgery, and that there were few technical conversions. This result suggests that referral to specialized institutions, such as high-volume centers and university hospitals, especially for patients exhibiting relevant background risk factors, may reduce the risk of conversion to laparotomy and lead to better outcomes for patients.

Some studies have reported that surgery by inexperienced or young surgeons is a risk factor for conversion to laparotomy [15, 19–22], whereas others have reported conflicting results [43]. In the present study, the lack of surgical participation of Japanese ESSQS-certified physicians was extracted as a risk factor for both indicated conversion and technical conversion. Numerous reports related to laparoscopic low anterior rectal resection have demonstrated that technical safety, surgical outcomes, and short-term postoperative outcomes improve when the surgery is conducted by an ESSQS-certified physician [44–48], which may explain the relatively low rate of technical conversion observed in the current study. Furthermore, our results suggest that even coaching or assistance by ESSQS-certified physicians can contribute to the safe implementation of laparoscopic procedures by decreasing the likelihood of technical conversion. Accordingly, decreases in the rate of indicated conversion may be explained by the increased accuracy of preoperative predictions and surgical indications when made by or in conjunction with ESSQS-certified physicians.

Several limitations associated with the present study warrant mention. First, its retrospective, non-randomized design may have resulted in selection bias. Since the data were collected retrospectively, medical history such as laparotomy history that may have related to the presence of adhesions could not be collected. Second, the study population was obtained from institutions included in the JSLCS, which may have introduced bias given the high number of surgeries in which ESSQS-certified physicians participated. In the present study, over 80% of surgeries involved the participation of ESSQS-certified physicians. However, in actual clinical practice, ESSQS-certified physicians account for less than 10% of the general surgeon population in Japan [30]. Research using the National Clinical Database (NCD) found that ESSQS-certified surgeons conducted approximately 30% of all laparoscopic low anterior resections between 2014 and 2016 [44]. As the NCD does not indicate whether ESSQS-certified physicians participated in surgery as assistants or instructors, the exact participation rate is unknown, although it is predicted to be lower than that in our study. Increasing the number of cases without the participation of an ESSQS-certified physician may more closely approximate the characteristics of actual clinical practice.

Despite these limitations, our analysis was based on accumulated data from a large number of patients with rectal cancer undergoing laparoscopic surgery across multiple institutions, enabling us to adjust for clinically significant biases through a multivariate analysis.

## Conclusions

Our results indicated that tumor diameter, combined resection of adjacent organs, and surgical non-participation of an ESSQS-certified physician are risk factors for indicated conversion to laparotomy. Similarly, registered case number, institution type, combined resection of adjacent organs, and surgical non-participation of an ESSQS-certified physician were identified as risk factors for technical conversion. These results suggest that participation of ESSQS-certified physicians may reduce the risk of both indicated and technical conversion. Notably, our results suggest that even coaching or assistance by ESSQS-certified physicians can contribute to the safe implementation of laparoscopic procedures. Necessary countermeasures for these risk factors should include an accurate preoperative diagnosis and a sufficient examination of indications as to whether or not laparoscopic surgery can be completed at individual institutions and by specific surgical members. Referral to specialized institutions, such as high-volume centers and university hospitals, especially for patients exhibiting relevant background risk factors, may reduce the risk of conversion to laparotomy and lead to better outcomes for patients.
